# Pure Water

**Published:** 1883-02

**Authors:** 


### PURE WATER.

There is nothing so essential to health and comfort as an abundant supply of pure, fresh water ; we partake of it more abundantly than we do of any other substance, since, in addition to that taken in various forms to allay thirst, whatever enters the stomach as food is largely saturated with it; indeed, four-fifths of the weight of our bodies is water. If the source of supply is contaminated by decayed vegetable matter, by cess-pools and sewage from defective drainage, which is more frequently the case than is generally believed, such water is certain sooner or later to induce disease.

Nature seems, in some unaccountable manner, to tolerate abuses for a long time ; hence it is one of the most difficult undertakings in the world to convince the average man that all well water is not pure and wholesome, even though his well may be filled to overflowing, after a heavy rain, with the washings from a barn yard. There are but few open wells throughout the country where the depth of water is not increased in proportion to the amount of rain-fall. Nearly all this increase consists of surface water that is impregnated with whatever the soil contains for a considerable distance around ; not to mention the drowned insects and worms that are frequently carried along in quantities sufficient to render the water putrid with their decaying bodies. I believe that this poisonous water supply, is the chief source of all forms of malarial fever, and that that *bête noir* called *miasma*, which is supposed to emanate from the decaying vegetation of marshy ground, but whose form and substance all the appliances of modern science have failed to detect, may have less to do with these forms of disease, than is generally supposed. I know of families that have resided near marsh lands, in a so-called "fever and ague district" for many years, that have never had fever and ague, while others supposed to be more favorably located have suffered greatly from the various types of this malady.

I have in several instances inquired into the causes of the immunity from sickness in one case, and it its causes in the other, and the. views here stated have been invariably confirmed.

I know of a house, located near a marsh, and where half the families in its vicinity suffer from malarial fevers.

Two families had occupied the house at different times, and both left on account of sickness. All the water they used was supplied from an open well, 10 feet deep, in the lowest portion of the door yard. A third family purchased the property for less than half its cost, made a " driven well" 25 feet deep, from which they get all the water used for drinking and cooking. They have occupied the premises over four years, and enjoy almost perfect health.

